# Patient Experiences With Prescription Cannabinoids in Germany: Protocol for a Mixed Methods, Exploratory, and Anonymous Web-Based Survey

**DOI:** 10.2196/38814

**Published:** 2023-03-21

**Authors:** Jan Moritz Fischer, Farid-Ihab Kandil, Matthias Karst, Laura Sophie Zager, Michael Jeitler, Felix Kugler, Franziska Fitzner, Andreas Michalsen, Christian S Kessler

**Affiliations:** 1 Institute of Social Medicine, Epidemiology and Health Economics Charité – Universitätsmedizin Berlin Corporate member of Freie Universität Berlin and Humboldt-Universität zu Berlin Berlin Germany; 2 Department of Anesthesiology and Intensive Care Medicine Pain Clinic Hannover Medical School Hannover Germany; 3 Department of Internal and Integrative Medicine Immanuel Hospital Berlin Berlin Germany

**Keywords:** cannabis, cannabinoids, representative survey, pain, mixed methods

## Abstract

**Background:**

Medical cannabinoids are controversial. Their use is comparatively rare, but it is rising. Since 2017, cannabinoids can be prescribed in Germany for a broader range of indications. Patient surveys on these drugs are hampered by the stigmatization of cannabinoids and their (still) low prevalence in medical contexts. Against this background, patients’ willingness to provide information is limited. Moreover, it is logistically challenging to reach them with a survey. A thorough knowledge of currently ongoing therapies and their effects and side effects, however, is important for a more appropriate and effective use of cannabinoids in the future.

**Objective:**

This study is an exploratory data collection using a representative sample. The main goal is to provide a detailed picture of the current use of medical cannabinoids in Germany. It is intended to identify subgroups that may benefit particularly well or poorly.

**Methods:**

We are conducting a representative, anonymous, cross-sectional, one-time, web-based survey based on mixed methods in 3 German federal states. Health conditions under cannabinoid therapy and before are documented with validated, symptom-specific questionnaires. This allows an estimation of the effect sizes of these therapies. The selection of parameters and questionnaires was based on the results of independent qualitative interviews in advance. Representative samples of the hard-to-reach study population are obtained by cluster sampling via contracted physicians of the statutory health insurance companies.

**Results:**

Recruitment was ongoing until the end of June 2022, with 256 enrolled participants. Validated questionnaires on pain, spasticity, anorexia or wasting, multiple sclerosis, nausea or vomiting, depression, and attention deficit hyperactivity disorder (ADHD) were selected. Symptom scores are being assessed for both current conditions under cannabinoid therapy and conditions prior to this therapy (in retrospect). Validated questionnaires are also used for treatment satisfaction and general quality of life. These are supplemented by existing diagnoses, a detailed medication history, any previous experiences with cannabis or illegal substances, experiences with the prescription process, and sociodemographic data. Based on the results of the previous qualitative interviews, questions were added regarding prior experience with relaxation methods and psychotherapy, personal opinions about cannabinoids, pre-existing or symptom-related psychological trauma, and different experiences with different cannabis-based therapies.

**Conclusions:**

The exploratory mixed methods approach of this project is expected to provide valid and relevant data as a basis for future clinical research. The study design may be representative for a large proportion of outpatients treated with cannabinoids in the German federal states studied. It may have less bias toward social desirability and may provide valuable information in addition to existing studies. Due to the observational and cross-sectional nature of this study, various limitations apply. Causal relations cannot be drawn.

**Trial Registration:**

German Clinical Trials Register DRKS00023344; https://drks.de/search/en/trial/DRKS00023344

**International Registered Report Identifier (IRRID):**

DERR1-10.2196/38814

## Introduction

### Background

Cannabis plants contain a variety of active ingredients. More than 100 cannabinoid compounds have been identified [1]. The most relevant for medical applications are tetrahydrocannabinol and cannabidiol [2]. Many different pharmacologically active cannabis ingredients exist, especially terpenes and terpenoids, and the synergistic effects of different compounds are being discussed widely [2,3]. Nevertheless, pure tetrahydrocannabinol and cannabidiol drugs are also frequently used. After the discovery of the endocannabinoid system in the 1990s, interest in medical applications of cannabinoid drugs has been increasing [2,4]. At the same time, cannabinoids are still controversial in most countries, including Germany, despite changing legal frameworks in medicine and society [5].

Proponents of cannabinoid medications tout their benefits for a variety of conditions [2,6-8]. Cannabinoid drugs are already used in the fields of pain management for multiple sclerosis, chronic pain syndromes, and cancer-related pain. Currently, 73% of cannabinoid prescriptions in Germany are for pain [9]. Previous studies show a substantial improvement in 35.5% and a moderate improvement in 34.2% of patients treated [10]. Other common indications include spasticity in multiple sclerosis, nausea in patients with cancer, and specific mental health symptoms [11,12].

Feared side effects are psychosis, addiction, and physical intolerance reactions [13,14]. This includes dizziness, nausea, fatigue, euphoria, disorientation, confusion, loss of balance, and hallucinations [15]. The risk of side effects is higher for older adults due to their impaired metabolism and comorbidities, as well as their increased potential for drug-to-drug interactions [16]. Side effects also vary depending on the medication used.

Due to legislative changes in Germany in 2017, a wider range of cannabinoid medications can be prescribed medically [17]. By law, physicians who prescribe medical cannabinoids at the expense of statutory health insurance companies were required to participate in a mandatory monitoring study for each patient until 2022 [9]. Parameters included indication, effectiveness, drug, dosage, and quality of life. All were evaluated solely by the prescribing physicians. Patients with private health insurance and those who paid out of pocket were not monitored. Most of the scientific evidence on the current state of cannabinoid therapies in Germany stems from this source only. There is no representative data from direct patient surveys.

Considering the risks and benefits, as well as the ongoing social controversy, a more accurate mapping of the current use of medical cannabinoids in Germany is desirable. There is a need for precise differentiation according to the underlying illnesses, illness-related restrictions, and the resulting health and psychosocial consequences, involving patients and not only their physicians [18]. Such findings could be valuable for a more targeted use of medical cannabinoids and could help in the formulation of questions for future clinical research in this field.

### Study Design and Goal

This is an anonymous, exploratory, cross-sectional, one-time, web-based survey using a mixed methods approach. All information is collected at the same point in time, past and current health status included. It aims to obtain representative, descriptive data on patients receiving outpatient treatment with medical cannabinoids in Germany and on their subjective treatment experiences. It is also intended to further explore the patient perspective of cannabis-based therapies and identify subgroups that may benefit particularly well or poorly.

### Qualitative Preliminary Study

Between September 2020 and March 2021, after approval by the local ethics committee (8391_BO-K_2019), a qualitative survey was conducted at the Pain Clinic at Hanover Medical School, Germany. Upon written consent, 32 patients (15 women) with chronic pain who had taken cannabis-based medicines for at least 6 months were deliberately selected as a purposive sample, according to Moser and Korstjens [19]. Interviews took 16-88 minutes (on average, 35 minutes). These were semistructured interviews with no time limit. The interview guideline had been constructed according to Kallio et al [20]. It consisted of an introduction that explained the purpose of the interview as well as the process. This was intended to promote an open and trusting interview atmosphere. This was followed by open-ended questions about previous experiences with cannabis and the effects on different areas of life. The interviews were recorded and later transcribed. The data was analyzed according to Strauss et al (grounded theory) [21]. Data collection continued until qualitative saturation occurred, which means that no new knowledge could be generated by further collection of data [22].

All participants were interviewed by the same researcher (FF), who also transcribed and analyzed the interviews. In addition, 3 selected interviews were independently analyzed by 3 other researchers (MK, MF, and FIK). Results were reviewed and discussed by the entire research team to achieve higher intercoder reliability.

The findings on the patient perspective of cannabinoid therapy were used as an additional basis for selecting outcome parameters and corresponding validated questionnaires. However, the detailed presentation of the analysis of these qualitative interviews is beyond the scope of this study and will be presented elsewhere.

The results led to questions about previous experiences with relaxation techniques or psychotherapy, as these methods are considered standard treatment for some of the reported symptoms [23-26]. The findings also resulted in the decision to inquire about the personal opinion about cannabinoids, preexisting or symptom-related psychological trauma, and about varying experiences with different cannabis-based medications.

## Methods

### Setting, Recruitment, and Sampling

We are surveying patients who receive prescription cannabinoids. The original target size is 300 participants. A representative sample for the German federal states of Berlin, Brandenburg, and Lower Saxony is drawn in a stratified procedure (see the *Data Collection, Management, and Analysis* section for details).

The web-based survey is conducted anonymously using mobile website technology. It starts with patient-adapted questions analogous to those in the mandatory monitoring survey. Based on this, further detailed information on the respective underlying disease is collected. The previous course of the disease and previous therapy attempts are asked in detail. In addition, information on cannabis use throughout the life course is requested, as well as on attitudes and opinions toward cannabinoids prior to the first prescription, where these stemmed from, and to what extent they have changed since the ongoing cannabinoid prescription.

### Sample Size

A total of 300 patients currently receiving cannabinoid therapy were to be included in this study (see Textbox 1). Based on the mandatory survey data, approximately 210 patients (69%) will likely experience pain symptoms [9]. Based on this assumption, a power analysis for this exploratory study indicates that using the standard parameters of *α*=0.05 and *β*=0.20 (corresponding to a power of 80%), *t* tests used for the pre-post comparisons among the 210 patients with pain symptoms can detect all large, medium, and even smaller effects with effect sizes of Cohen *d*≥0.20

**Eligibility criteria.**

**Inclusion criteria**

- Being 18 years or older
- Ongoing therapy with medically prescribed cannabinoids or therapy with medically prescribed cannabinoids in the 12 months prior to survey participation
- Stable internet access
- Have sufficient German language skills, as well as cognitive and physical abilities, to participate in a web-based survey in German, lasting approximately 30 minutes
- Active, written web-based consent (see Multimedia Appendix 1)

**Exclusion criteria**

- Lack of active, written web-based consent

### Outcome Parameters

#### Overview

The most common reasons for prescribing cannabinoids in Germany are known from the abovementioned mandatory survey. They are chronic pain (73%), spasticity (10%), anorexia or wasting (6%), multiple sclerosis (6%), nausea or vomiting (5%), depression (3%), and attention deficit hyperactivity disorder (ADHD; 1%) [9]. Accordingly, validated questionnaires on these conditions were selected for this survey. Symptom scores are being assessed for both current conditions under cannabinoid therapy and conditions prior to the cannabinoid therapy (in retrospect). Validated questionnaires are also used for treatment satisfaction (current and in retrospect) and for general quality of life (current and in retrospect). These are supplemented by existing diagnoses, a detailed medication history, any previous experiences with cannabis or illegal substances, experiences with the prescription process, and sociodemographic data. Modifications and additions were made based on the results of the qualitative interviews (see the *Qualitative Preliminary Study* section).

#### Treatment Satisfaction Questionnaire for Medication Version II

The Treatment Satisfaction Questionnaire for Medication (TSQM) version II is a general measure of patients’ satisfaction with their medication [27]. It is viewed as a reliable and valid instrument consisting of 4 scales: side effects, effectiveness, convenience, and global satisfaction [27-29].

#### Patient-Reported Outcome Measurement Information Systems

The Patient-Reported Outcome Measurement Information Systems (PROMIS-29) represents an international standard for the evaluation of quality of life based on a patient report. This test consists of 29 questions about the domains of physical function, anxiety, depression, fatigue, sleep disturbance, the ability to participate in social roles and activities, pain interference, and pain intensity [30,31].

#### Face Pain Scale

The Face Pain Scale represents an intuitive and well-established way to assess pain. It has been validated for both children and adults in various contexts [32,33].

#### Multiple Sclerosis Spasticity Scale 88

Spasticity is assessed using the Multiple Sclerosis Spasticity Scale 88 [34]. Due to its considerable length, we decided to only include the 4 most clinically relevant subscales in the survey: muscle stiffness, muscle spasticity, walking, and physical movement.

#### Anorexia Nervosa Inventory for Self-Rating

For patients experiencing anorexia, we use the Anorexia Nervosa Inventory for Self-Rating which includes questions about figure consciousness, feeling of insufficiency, anancasm, adverse effect of meals, sexual anxieties, as well as bulimia [35].

#### Minimal Documentation System on Distressing Symptoms

The Minimal Documentation System on Distressing Symptoms (MIDOS^2^) is a short questionnaire for non–anorexia-mediated weight loss and nausea or vomiting. This test inquires about a few symptoms associated with palliative care patients as well as their general condition and was validated for this patient group. Since the addressed items are not specific to palliative care, we expect MIDOS^2^ to allow an adequate evaluation of the examined symptoms [36].

#### Hospital Anxiety and Depression Scale

For patients with depression, the Hospital Anxiety and Depression Scale is applied. This test, comprising 14 questions, is a very common, international instrument for quantifying anxiety or depression [37-39]. The Hospital Anxiety and Depression Scale can be used to track disease progression and response to psychotherapeutic and psychopharmacological interventions [40].

#### Adult ADHD Self-Report Scale

The Adult ADHD Self-Report Scale version 1.1 assesses ADHD and was originally developed in cooperation with the World Health Organization and the Workgroup on Adult ADHD. It consists of 2 parts, the first of which inquires about inattentiveness and the second of which evaluates hyperactivity and impulsivity [41,42].

#### Rights of Use of the Applied Questionnaires

The use of the validated questionnaires for academic purposes required the consent of the parties holding the copyright. For TSQM, PROMIS-29, and Multiple Sclerosis Spasticity Scale 88, licenses had to be obtained. The authors of the remaining questionnaires required proper referencing.

### Data Collection, Management, and Analysis

A stratified random sample is collected via the prescribing contract physicians of the statutory health insurance companies. (88.1% of the German population has statutory health insurance [43]). All physicians with statutory health insurance contracts, medical specialist titles in anesthesiology, general medicine, neurology, and internal medicine, and who practice in the 3 federal states of Berlin, Brandenburg, and Lower Saxony are selected. According to the aforementioned mandatory monitoring survey, those 4 groups of specialists together account for 88% of prescriptions for cannabinoids in Germany [9].

Complete lists of these physicians are obtained by a formal request for academic research purposes from the corresponding medical councils at the federal state level. All physicians on those lists are contacted via email or, in the case of Lower Saxony, via fax (in Lower Saxony, email addresses could not be provided). In addition, physicians are contacted by telephone in random order and asked to invite their own patients who receive cannabinoid medication to partake in the survey, outpatient tertiary centers included. Randomization is done by random number values without duplicates using Microsoft Excel. In the event of unavailability, 3 attempts are made at different times. In the meantime, the next physicians on the list are called. This continues until the planned number of participants is reached.

Physicians who wish to forward study invitations to their patients send an email to the study secretariat requesting an appropriate number of anonymous study invitations. They can then forward the invitations either via email or as a printout to their patients. Each study invitation contains a unique participation code, which serves as the login code for the web-based survey. The study center never receives any personalized or pseudonymized patient data. In this way, a fully anonymous and representative survey is made possible. Both physicians and participants receive an incentive for participation. Physicians receive €50 (US $60) per patient of theirs who participates in the web-based survey as compensation for their time. Since it is known which physicians have received which codes to pass on to their patients, the compensation can be calculated. Information about which physician received which codes is strictly separated from the study data. The complete anonymity of the study participants is thus preserved. On the last page of the web-based survey, patients are shown an impersonalized voucher code worth €15 (US $18) for books from a German bookstore.

The web-based survey is conducted using LimeSurvey (version 5.1.10; Carsten Schmitz) running on a dedicated and secure local server at Charité—Universitätsmedizin Berlin. Entries made by the individual subjects are checked for plausibility and completeness, where possible. Possibly missing data will be imputed using the Multiple Imputation by Chained Equations algorithm [44]. Clearly implausible or very incomplete entries (>50% missing data) will be excluded from the analysis.

Data from this exploratory cross-sectional study will be evaluated primarily using descriptive statistics. Parametrical figures will be provided, as well as risk and odds ratios for selected questions. Scores for all questionnaires are obtained twice from the participants in the same session. They are first instructed to answer the questionnaires based on their current state, and then on what they remember their state being like prior to the commencement of the cannabinoid therapy. Individual *t* tests will be applied to assess, on a strictly exploratory level, whether within-group changes constitute a substantial improvement. This assessment will rely on both significance levels and effect sizes (Cohen *d*). As there is no control group, findings will be interpreted with due precaution. Furthermore, as a sensitivity analysis, the results for the main tests for pain (numeric analog scale), treatment satisfaction (TSQM), and quality of life (World Health Organization-5 Well-Being Index) will be tested for confounders by extending the *t* test to ANOVA and analysis of covariance models. The authors are very aware of the limited informative value of a pre-post assessment as part of a one-time survey in an observational study design, especially with regard to causal attributions.

The data is stored at the Charité—Universitätsmedizin Berlin for 10 years before being deleted. Only the responsible staff of the study center has access. There is continuous monitoring of enrollment during the survey phase. This is necessary to reach the planned number of participants. If necessary, slow recruitment can be compensated for by contacting more physicians on the phone (this is in addition to contacting all physicians via email or fax). Phone calls are carried out by the responsible study physician, trained study nurses, and trained student assistants. Additional student assistants are available if speeding up recruitment is required. Monitoring of enrollment status is performed by the responsible study team. No data monitoring committee is required because this is a noninterventional observational study. No interim analysis of the data is planned.

### Ethics Approval

The ethics committee of the Charité—Universitätsmedizin Berlin gave its approval to obtain, process, and analyze the data and to publish the results (German Clinical Trials Register DRKS00023344; ethics number EA1/327/20). All participants provided their informed written web-based consent to participate in the study. The study is conducted in accordance with the Declaration of Helsinki in its currently valid version, the guidelines of the International Conference on Harmonization of Good Clinical Practice, and the applicable German laws.

### Patient and Public Involvement

Patients are not involved in the design, implementation, reporting, or dissemination plans of our research. Yet, they are a key element in the choice of parameters via qualitative interviews. All parameters in the survey are patient-reported parameters.

### Timeline

**Figure 1:**
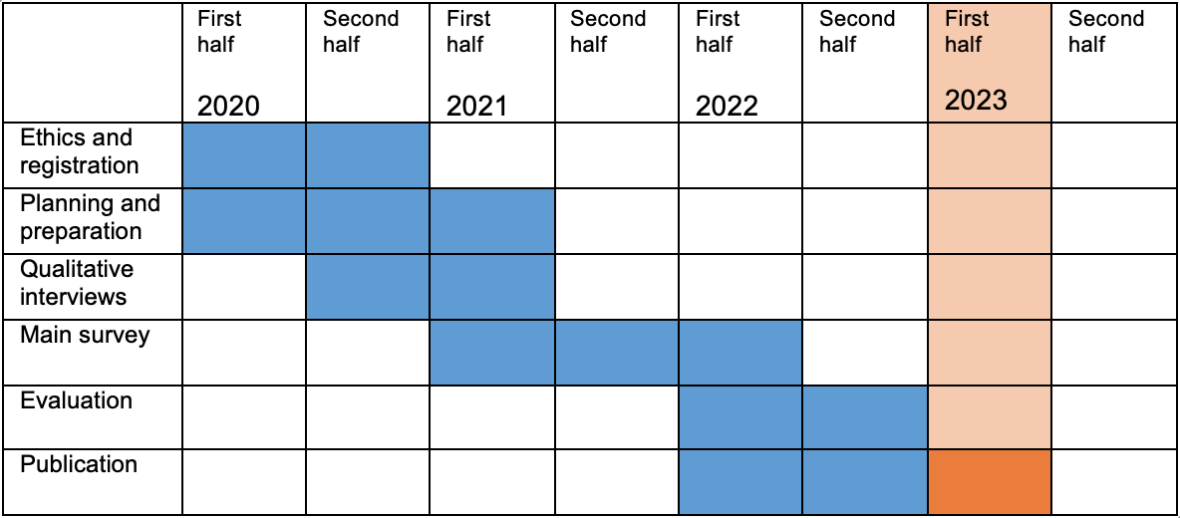
Timeline.

The timeline of this study is presented in Figure 1.

## Results

Recruitment was ongoing until the end of June 2022. A total of 11,744 physicians were contacted to invite their patients who receive cannabinoid medication to participate in the study (Berlin, n=1700; Brandenburg, n=2041; Lower Saxony, n=8003). At the time of submission, 256 participants had been enrolled. The COVID-19 pandemic markedly slowed down participant enrollment. Many physicians did not have the capacity to forward the study invitation to their patients as they had to cope with unusually high numbers of patients, sick leave among their own staff, and other extra duties. We were frequently told that while there was interest in principle in supporting the study, the pandemic conditions at the time did not allow for activities outside of regular patient care. Due to slow recruitment, the survey was later expanded to the federal state of Brandenburg (amendment approved by the ethics committee on January 20, 2022.) Here, all physicians were contacted once by email. Direct calling in random order could not be carried out in Brandenburg before the study was terminated. Recruitment was terminated as no further funding was available to extend the recruitment period. Results will be published in summer 2023.

## Discussion

### Principal Findings

We are conducting a one-time cross-sectional web-based survey of a cohort of patients receiving prescription cannabinoids in Germany. In addition, based on the results of a qualitative preliminary study, a clinically relevant selection of parameters was made for the study presented here. In addition to data on quality of life, psychological parameters (including anxiety and depression), and social participation over the course of cannabinoid treatment, we included questions on previous experiences with psychotherapy and relaxation procedures. We also included screening questions for trauma to detect differences in prevalence compared to the general population. While in the preceding qualitative interviews the effects of different cannabis-based preparations and doses were reported very differently (eg, from positive to negative by the same patient), we included questions on the cannabis-based preparations used and their respective perceived effects in their exact time course. This might help identify favorable dose ranges for different diagnoses and age groups based on patient experiences. The use of symptom-specific standardized questionnaires will also allow us to estimate effect sizes of cannabinoid therapies, while bearing in mind all present limitations within the scope of this study design. This survey collects detailed information on the patients’ underlying diseases, previous treatment attempts, and individual attitudes toward cannabis.

A strength of this study is its sample of a rather hard-to-reach study population in Germany and Europe using a representative approach. Cannabis consumption is stigmatized in parts of German society [45]. The use of medical cannabinoids is also still rare in Germany (approximately 27,000 patients in a total population of 83.2 million) [9]. This study may represent up to 77.5% of total cannabinoid prescriptions in Germany. The proportion of the total population of outpatients who receive cannabinoid medications in Germany, which is depicted in this study, has been estimated as follows: proportion of all patients with statutory health insurance (who are accordingly treated by physicians licensed by the statutory health insurance) multiplied by the proportion of these patients who are treated by the specialist groups involved in this study, 88.1% (73.3 million/83.2 million) [43] × 88% (8809/10010) [9] = 77.5%. In comparison, the mandatory monitoring survey was able to generate 9000 complete records out of at least 27,000 reported prescriptions (33.3%) [9]. Our intention is to supplement the existing data with a sample that significantly adds to it by involving the patient perspective.

The study design also provides a substantial reduction in social desirability bias due to the full anonymity of participants. The existing mandatory monitoring survey is directed at prescribing physicians who may have an interest in justifying their own therapeutic approach. When patients are surveyed by their prescribing physicians, they too may be biased by social desirability.

In contrast, social desirability bias is potentially lower in this study since it is anonymous. It is also more independent from prescribing physicians, as they merely forward the invitation, and patients fill out the questionnaire without the doctor’s presence at home. Lastly, the mixed method approach of this design is intended to provide especially valid and relevant data.

Nonetheless, there are several factors that may influence the answers in the questionnaires, like the doctor-patient relationship and the high patient compliance required for detailed data entry. The study’s major limitations also include a risk of selection bias. The web-based survey will most likely underrepresent or not represent certain patient groups, such as patients who are very old or very sick and are not able to participate in a web-based survey. These represent a relevant proportion of the total prescriptions for cannabis-based medicines in Germany. Wasting (and anorexia) and nausea in patients with cancer are among the most common reasons for prescribing, at 6% (641/10.010) and 5% (511/10.010), respectively [9]. The results of this study will only be transferable to them to a limited extent.

There is a considerable risk of recall bias. We try to capture a large amount of information in a cross-sectional survey. Some pieces of information may relate to long-past events. Attempts are made to mitigate this by indicating in both the study invitation and the virtual login area that it may take up to an hour to complete the web-based survey. Participants are also asked to have all available medical records ready and to complete the questionnaire in a quiet place. Nevertheless, the validity of the results will, of course, not be comparable to those of a prospective study.

There is no absolute control over whether individuals completing the web-based survey are actually patients who receive cannabinoid medication. Invitations and login codes can theoretically be given to third parties to receive an incentive (a €15 (US $18) voucher code for books). We assume an extremely low rate of abusive participation but cannot completely exclude it, making it a minor limitation to the validity of this study.

Up until now, about half of the randomly selected doctors could be successfully called on the phone and were asked to invite patients to participate. All doctors did receive an invitation by either email (Berlin and Brandenburg) or fax (Lower Saxony). This creates a risk for systematic bias in the sample.

It is quite certain that the groups of specialists included in the study were affected by the COVID-19 pandemic to varying degrees. Thus, for example, a higher workload in general medicine could lead to an underrepresentation of patients treated there. Medical practices that were particularly affected by the pandemic were significantly less likely to invite patients to participate in the study, and vice versa. This is understandable, as they did not have time to attend to matters beyond regular patient care. For this reason, a risk of sample bias remains.

The qualitative interviews of the independently performed previous study were held, recorded, transcribed, and analyzed by a single researcher. Only 3 out of 32 interviews were interpreted independently by other researchers for intercoder reliability. The large number of interviews conducted until theoretical saturation was reached may reflect the individual diversity of cannabinoid therapies and how they are experienced. It could also be an indication of methodological uncertainties in conducting the qualitative interviews. However, coding and results were discussed among the entire research team, including 2 experienced qualitative researchers. There was unanimity regarding the data and its interpretation.

Finally, it must be said that all the usual restrictions apply with regard to the interpretability of data in the context of cross-sectional, observational studies. Accordingly, the results must then be discussed with all due restraint and contextualized in a reflective manner.

### Conclusions

This study may contribute to a more comprehensive picture of experiences with medical cannabinoids from the perspective of patients with various diseases. Additional understanding of the risks and benefits of cannabinoid therapies may contribute to more appropriate use of these drugs and less societal stigma.

## Appendix

Multimedia Appendix 1 Digital privacy and consent statement for participation in an anonymous survey as part of the EXCARP study

## Abbreviations

ADHD: attention-deficit/hyperactivity disorder
MIDOS: minimal documentation system on distressing symptoms
PROMIS: patient-reported outcome measurement information system
TSQM: Treatment Satisfaction Questionnaire for Medication

## Data Availability

In accordance with the granted ethics approval, the study data will not be made publicly available to protect participant data. The data sets can be requested from the corresponding author upon justified request.
