# Supplementary material for: Patient Experiences With Prescription Cannabinoids in Germany: Protocol for a Mixed Methods, Exploratory, and Anonymous Web-Based Survey
Source: JMIR Res Protoc. 2023 Mar 21;12:e38814. doi: 10.2196/38814 (PMC10131879; doi:10.2196/38814)
Supplement: Multimedia Appendix 1 [file resprot_v12i1e38814_app1.docx]

**Appendix**

**Digital privacy and consent statement for participation in an anonymous survey as part of the EXCARP study**

With the following consent form, you give your consent to participate in the anonymous survey and to the scientific analysis of your answers. The consent form covers the following points:

- Background and motivation of the study
- Nature and objectives of the study
- Conduct of the study
- Voluntariness of participation and right to withdraw from the study
- Data protection: documentation, transfer and publication of data in anonymous form only. Rights of data subjects, right to have personal data deleted and competent bodies for complaints

Regardless of the information you provide in the survey, participation will in no way result in any disadvantage for you. In particular, participation will not lead to a change in your medication or any other intervention in your therapy.

**Background and motivation of the study**

Since the "Amendment of Annex III" of the Narcotics Act (BtMG) in 2011 and the "Act Amending Narcotics Law and Other Regulations" in 2017, cannabinoid medicinal products are increasingly available to more patients in Germany as prescription drugs. This fact allows to investigate even more precisely than before how cannabinoid medicinal products work in different clinical pictures. In particular, it is of interest whether certain patient groups can be identified that particularly benefit from therapy with cannabinoid medicines. In addition, any rare problems with cannabinoid therapy that may have been previously unknown can be recorded.

**Nature and objectives of the study**

This is a one-time and anonymous online survey. The questions refer partly to the present and partly to the past. The objective is to obtain as accurate a scientific understanding as possible of the cannabinoid therapies currently being conducted in Berlin, Brandenburg, and Lower Saxony. Of particular interest is a possible difference of these therapies to any treatments with other agents in the past.

**Conduct of the study**

The survey consists of a comprehensive online questionnaire that is completed completely anonymously. This means that you do not provide any personal information (e.g. name, date of birth, address) when filling out the questionnaire. Among other things, the questionnaire is made up of various validated test questionnaires. The individual test questionnaires allow, for example, the assessment of the severity of symptoms. A list of the individual test questionnaires can be found below.

Completing the entire questionnaire takes about 45 minutes. In individual cases, it may take shorter or longer. You may take breaks while completing it.

The survey asks about selected demographic variables (including age, occupation, marital status, education, sick leave in the past few months).

The following areas are also asked about:

- Quality of life
- Pain
- Pain consequences
- Therapy satisfaction
- Traumatization
- Illnesses (including symptoms, severity, hospitalizations, duration)
- Experience with cannabis/cannabinoids
- Inquiry about dietary habits
- Consumption behavior (e.g. alcohol, cigarettes)
- Inquiry about sports activity
- Inquiry about stays in nature
- Use of other treatment methods

**Voluntariness of participation and right to withdraw from the study participation**

Participation in this survey is completely voluntary. You are free to withdraw from the survey at any time - without giving any reason. This will not result in any consequences or disadvantages for you.

**Privacy policy**

The data collection is carried out by the University Outpatient Clinic for Naturopathy of the Charité University Medicine Berlin.  Your personal data is processed on the basis of the EU General Data Protection Regulation and the Berlin State Data Protection Act. The study director is the data controller within the meaning of the EU General Data Protection Regulation.

**Collection and storage of personal data as well as type and purpose of their use**

The content of the scientific survey is completely anonymous. All data are stored exclusively on specially secured servers of the Charité. They are therefore safe from theft and will under no circumstances be passed on to third parties not listed in this declaration.

When you visit this website, information is automatically sent to the server by the browser used on your terminal device. This information is stored temporarily in a so-called log file exclusively for technical reasons. The following information is collected without your intervention and stored until automated deletion:

- Anonymized (= shortened) IP address of the requesting computer,
- Date and time of access,
- Name and URL of the accessed file,
- Website from which the access was made (referrer URL),
- browser used and, if applicable, the operating system of your computer as well as the name of your access provider.

The aforementioned data is processed for the following purposes:

- Ensuring a smooth connection setup of the websites,
- Ensuring comfortable use of the website
- evaluation of system security and stability as well as
- for other administrative purposes.

The legal basis for the data processing is Art. 6 para. 1 p. 1 lit. f DSGVO. The legitimate interest of Charité follows from the purposes listed above exclusively for data collection in the context of the survey described above. In no case will the collected data be used for the purpose of drawing conclusions about your person.

**Publication of data**

Please note that the results of the study may be published in scientific literature, but your identity will remain anonymous in all cases.

**Your rights in connection with the survey**

You have the right to:

1. To request information about your processed personal data in accordance with Article 15 of the GDPR. In particular, you can request information about the processing purposes, the planned storage period, the existence of a right to rectification, erasure, restriction of processing or objection, the existence of a right of appeal, the origin of your data, as well as the existence of automated decision-making, including profiling, and, if applicable, meaningful information about its details;
2. pursuant to Art. 16 DSGVO, to demand the correction of incorrect or completion of your stored personal data without undue delay;
3. pursuant to Art. 17 DSGVO, to demand the deletion of your stored personal data.
4. In accordance with Art. 18 DSGVO, to request the restriction of the processing of your personal data, insofar as the accuracy of the data is disputed by you, the processing is unlawful, but you object to its erasure and we no longer need the data, but you need it for the assertion, exercise or defense of legal claims or you have objected to the processing in accordance with Art. 21 DSGVO;
5. pursuant to Art. 20 DSGVO, to receive your personal data that you have provided in a structured, common and machine-readable format or to request that it be transferred to another controller;
6. in accordance with Art. 7 (3) DSGVO, to revoke your consent once given at any time. This has the consequence that we may no longer continue the data processing based on this consent for the future.
7. complain to a supervisory authority in accordance with Art. 77 DSGVO. If you have any concerns about data processing and compliance with data protection requirements, you can contact the Data Protection Unit of Charité Berlin:

Data Protection Unit

Charitéplatz 1, 10117 Berlin

Tel: 030-450580016

E-mail: [datenschutz@charite.de](mailto:datenschutz@charite.de)

If you believe that your student data is being used in violation of applicable data protection laws, you may contact the Berlin Commissioner for Data Protection and Freedom of Information:

Berlin Commissioner for Data Protection and Freedom of Information

Friedrichstrasse 219

Tel: 030-13889-0

Fax: 030-2155050

E-mail: [mailbox@datenschutz-berlin.de](mailto:mailbox@datenschutz-berlin.de)

**Storage period**

Your data will be stored in accordance with the applicable legal requirements for scientific data in Germany, in completely anonymized form, for 10 years and then deleted.

**Persons involved**

Project management:

Prof. Dr. med. Andreas Michalsen

Endowed Chair for Clinical Naturopathy

Charité University Outpatient Clinic for Naturopathy at Immanuel Hospital Berlin

Königstrasse 63, 14109 Berlin

Tel: 030-80505 691

Fax: 030-80505 692

E-mail: andreas.michalsen@charite.de

PD Dr. med. Christian Kessler

Charité University Outpatient Clinic for Naturopathy at Immanuel Hospital Berlin-Wannsee

Königstraße 63, 14109 Berlin

Tel.: ++49 (0)30 - 80505 617

Fax: ++49 (0)30 - 80505 690/-692

E-mail: christian.kessler@charite.de

Prof. Dr. med. Matthias Karst

Outpatient Pain Clinic of the Hanover Medical School

Carl-Neuberg-Strasse 1, 30625 Hanover, Germany

E-mail: karst.matthias@mh-hannover.de

Project Coordination:

Jan Moritz Fischer

Charité University Outpatient Clinic for Naturopathy at Immanuel Hospital Berlin-Wannsee

Königstr. 63, 14109 Berlin

Tel.: ++49 (0)30 - 80505 682

Fax: ++49 (0)30 - 80505 690/-692

E-mail: [janmoritz.fischer@immanuelalbertinen.de](mailto:janmoritz.fischer@immanuelalbertinen.de)

Dr. med. Michael Jeitler

Charité University Outpatient Clinic for Naturopathy at Immanuel Hospital Berlin-Wannsee

Königstraße 63, 14109 Berlin

Tel.: ++49 (0)30 - 80505 682

Fax: ++49 (0)30 - 80505 690/-692

E-mail: michael.jeitler@charite.de

Statistics:

Dr. rer. nat. Farid-Ihab Kandil

Immanuel Hospital Berlin Wannsee,

Department of Naturopathy

Königstrasse 63

14109 Berlin

Phone: ++49 (0)30 - 80505 691

Fax: ++49 (0)30 - 80505 690/-692

E-mail: farid-ihab.kandil@charite.de

**Qualitative**

1. Who initiated the therapy with a cannabis drug?
2. If the initiative for therapy with a cannabis drug came from you:
3. By what was your initiative justified?

Give examples, if applicable:

Media reports, own experience with non-medical cannabis.

1. How costly was the path to treatment for you?
2. Why was the cannabis medicine prescribed to you?
3. What did you expect from the cannabis medicine when it was prescribed to you?
4. To what extent have your hopes been fulfilled?
5. To what extent have your hopes not been fulfilled?
6. What has changed for you since you started taking the cannabis medicine?
7. How would you describe the effects of the cannabis medicine?

ask if necessary:

Stress, tension, psychological distress, difficulty falling asleep/staying asleep, appetite.

1. If you have prior experience with non-medical cannabis:
   What is the difference?
2. If you have used multiple cannabis medicines, how would you describe the differences in their effect on your symptoms?
3. What impact does this have on your daily life?
4. How and in what ways has your quality of life changed since you started cannabis medicine therapy?
5. You are a patient at the pain outpatient clinic. How does the effect of the cannabis medicine affect your pain?
6. Is there anything else you would like to say about this topic?

**Revised Interview guide**

1. Who initiated the therapy with a cannabis drug?
   1. If the initiative for therapy with a cannabis drug came from you:
      1. By what was your initiative justified?

Give examples, if applicable: Media reports, own experience with non-medical cannabis.

- - 1. How costly was the path to treatment for you and which experiences did you make with persons involved in the health care system?

Examples: physicians, pharmacists, health insurance, police

1. Why was the cannabis medicine prescribed to you?
2. What did you expect from the cannabis medicine when it was prescribed to you?
3. To what extent have your hopes been fulfilled respectively not been fulfilled?
4. What has changed for you since you started taking the cannabis medicine?
5. How would you describe the effects of the cannabis medicine?

ask if necessary:

Stress, tension, psychological distress, difficulty falling asleep/staying asleep, appetite.

1. If you have prior experience with non-medical cannabis:
   What is the difference?
2. If you have used multiple cannabis medicines, how would you describe the differences in their effect on your symptoms?
3. What impact does this have on your daily life?
4. How and in what ways has your quality of life changed since you started cannabis medicine therapy?
5. You are a patient at the pain outpatient clinic. How does the effect of the cannabis medicine affect your pain?
6. To what extent would you say that you generally find it difficult to relax or switch off, also independent from pain?

Examples: from work, tasks to be conducted and goals

- 1. What role does cannabis therapy play in this context?

1. Have you ever received support from psychological or behavioral therapy in connection with your pain disorder?

Examples: talk therapy, PMR according to Jakobson, mediation, autogenic training, yoga

- 1. How did this help you?
  2. What parallels do you see between these procedures and the effects of cannabis medicine?

1. Is there anything else you would like to say about this topic?
